# Prevalence and Factors of Anxiety and Depression in Chronic Kidney Disease Patients Undergoing Hemodialysis: A Cross-sectional Single-Center Study in Saudi Arabia

**DOI:** 10.7759/cureus.6668

**Published:** 2020-01-15

**Authors:** Hanan Mosleh, Meaad Alenezi, Samah Al johani, Arwa Alsani, Ghadeer Fairaq, Reenad Bedaiwi

**Affiliations:** 1 Family and Community Medicine, College of Medicine, Taibah University, Madinah, SAU; 2 Family Medicine, College of Medicine, Taibah University, Madinah, SAU

**Keywords:** chronic kidney disease, depression and anxiety, hemodialysis, end stage kidney disease

## Abstract

Introduction

Mood disorders, including anxiety and depression, are prevalent among patients with chronic kidney disease (CKD) who are on hemodialysis. Anxiety and\or depressive symptoms among those patients have been associated with early initiation of dialysis and adverse outcome.

Aim

The aim is to investigate the prevalence and factors associated with anxiety and depression among Saudi patients with CKD who are on hemodialysis.

Methods

This is a cross-sectional study. A total of 122 patients with CKD and on hemodialysis at King Fahad Hospital in Al-Madinah, Saudi Arabia, were included in the study during the period from November 2017 to August 2018. Data were collected using the Hospital Anxiety and Depression Scale questionnaire. Sociodemographic information, duration of illness, and duration of hemodialysis were determined.

Results

Of the 122 CKD patients, 24.6% had depression and 19.7% had anxiety symptoms. Anxiety symptoms were more prevalent among females than males (P = 0.04). Older age was significantly associated with depression (P = 0.003). Patients’ depression and anxiety symptoms were insignificantly associated with their education level, employment status, duration of illness, and duration of hemodialysis.

Conclusion

Anxiety and depression are prevalent among CKD patients, particularly among females and older patients. Thus, this study suggests establishing a screening program to determine patients who are at risk of developing anxiety and depression. In addition, management to prevent the occurrence of depression and anxiety and improve patients’ quality of life must be implemented.

## Introduction

Chronic kidney disease (CKD) is a complex condition in which the kidneys are unable to function properly as a result of structural or functional damage that leads to excessive fluid and waste accumulation in the blood [1]. CKD represents a major economic burden on healthcare systems worldwide [2]. Nowadays, the prevalence of CKD is rising significantly. The estimated number of affected people ranges from 11% to 13% globally [3]. The incidence and prevalence of CKD have increased rapidly among the Saudi population in the last three decades. However, research has yet to find out the exact number of people in each stage of CKD in Saudi Arabia. There is an obvious rise in the incidence and prevalence of CKD in all areas of Saudi Arabia, especially in the Western region when compared with other regions due to multicultural community. The incidence of diabetes mellitus in Saudi Arabia as the main cause of CKD ranges from 12% to 26%, followed by hypertension, which affects approximately 25% of the adult population [4].

It is well known that CKD is associated with age-related kidney function descent commonly exacerbated by diabetes, hypertension, and obesity. CKD can be categorized into five stages based on glomerular filtration rate (GFR). The first stage is defined as GFR below 60 mL/minute/1.73 m^2^, which is considered abnormal in all age groups. In contrast, end-stage kidney disease, or stage V CKD, is the most severe form of CKD because the kidneys are unable to effectively maintain homeostasis [5]. From diagnosis, CKD patients need to be treated for the rest of their lives; however, the emotional and psychological aspects of the patients’ disease are usually overlooked in everyday medical practice. While most physicians are aware of this reality, they usually have little time to effectively assess and address patients’ emotional states.

Depression is an important issue that healthcare professionals have to look after. Much research confirms a high prevalence of depression and anxiety among patients with CKD. It is estimated that 23.7% of patients with CKD have depression. Additionally, CKD patients on dialysis are more likely to develop depression (34.5%) compared with patients not on dialysis (13.3%) [6].

Regarding the correlation of depression with CKD, two cross-sectional studies reported a higher prevalence of depression among CKD patients who reported having no religious beliefs, followed no regular exercise regimen, had sleep disorders, and were diagnosed with stage III or above CKD. Both of those studies agree that there is a significant correlation between the stage of the kidney disease and depression: those with advanced CKD, i.e., stage III and above, are more prone to depression; however, one of those studies correlates depression particularly in elderly men with stage III-V CKD but it found no significant change among women. In general, however, the prevalence of depression is higher among women regardless of CKD stage; the female-to-male prevalence ratio for depression is widely accepted to be approximately 2:1 [7-8].

The aim of this study is to determine the prevalence of anxiety and depression among CKD patients undergoing hemodialysis. Thus, the findings of this study will serve as a basis to initiate a needs assessment among CKD patients experiencing anxiety and depression and to develop and implement a support management plan to improve mental health services for these CKD patients and improve their quality of life.

## Materials and methods

Study design

This is a cross-sectional study with an analytical component.

Study setting and study period

The study was conducted at King Fahad Hospital Dialysis Unit in Al-Madinah, Saudi Arabia, during the period from November 2017 to August 2018.

Study participants, sampling design, and sample size

Patients were recruited from the list of patients appointed to the dialysis unit. The inclusion criteria for the participants were as follows: Saudi, age ≥ 18 years, and CKD patient undergoing hemodialysis. The exclusion criteria were as follows: non-Saudi, CKD patients who are treated by modalities other than hemodialysis, and patients undergoing hemodialysis outside the study setting.

Sample size was calculated using the OpenEpi online sample size calculator using the sampling equation for a finite population: sample size n = DEFF*Np(1-p)]/ [(d2/Z21-α/2*(N-1)+p*(1-p) [9].

The total number of patients registered in the dialysis unit was 260, of which 220 on hemodialysis and 40 were on peritoneal dialysis. Inputs entered were a total population size of 220 CKD patients attending the hemodialysis unit (finite population), a hypothesized frequency of depression of 22.6% and at 5% precision, and a confidence level of 95%. These inputs yielded a sample size of at least 122 participants.

Data collection

*The data collection tool consists of the following* *three sections:*

1. Personal and sociodemographic information (i.e., age, sex, residence, patient’s education level, and employment status).

2. Disease-related information: date of diagnosis with CKD, duration of hemodialysis, number of hemodialysis sessions per week, and duration of each session.

3. An Arabic-language version of the Hospital Anxiety and Depression Scale (HADS) questionnaire was self-administered to the patients. The HADS questionnaire is a tool designed to measure anxiety and depression with two independent subscales. The elements for all the parts are measured and are rated on a four-point Likert-type scale (from 0 to 3).

Validity

This Arabic version of the HADS questionnaire has been validated among hospitalized patients in two different tertiary centers and in primary care settings in three Gulf countries: Saudi Arabia, Kuwait, and the United Arab Emirates. Its validity was examined by assessing construct validity by investigating the correlations between different validated depression and anxiety measures including the Generalized Anxiety Disorder 7-item scale (GAD-7) and Major Depression Inventory (MDI) scale, in addition to face validity (in which the patients were questioned about their perspectives on the HADS questionnaire). As regards construct validity, there was a strong association between HADS and GAD-7 regarding anxiety scores and strong associations between HADS and MDI in relation to depression scores. Regarding face validity, the vast majority of patients faced no difficulties in understanding the questions given in the assessment; they also reported that it covered most of the aspects entailed in their hospital anxiety and depression and thus they agreed that they would like to use this tool in the future for further assessment as it did not breach their privacy but did address important points regarding their pain [10].

Reliability

Its reliability was assessed twice among patients and on two time periods (time 1 and time 2). Cronbach’s alphas for the HADS anxiety subscale were 0.83 (95% confidence interval [CI]: 0.79-0.88) and 0.87 (95% CI: 0.83-0.91) for time 1 and time 2, respectively. Cronbach’s alphas for the HADS depression subscale were 0.77 (95% CI: 0.7-0.83) and 0.8 (95% CI: 0.75-0.86) for time 1 and time 2, respectively. This shows adequate internal consistency for both time points [10].

Outcome measures

The primary outcome of this study is to estimate the prevalence of depression and anxiety among CKD patients who are undergoing hemodialysis. According to the HADS questionnaire, individuals with a score of 7 or below are considered to be normal, individuals with a score of 8-10 are considered to be on the borderline of depression and anxiety, and individuals with a score of 11-21 are considered to be depressed and have anxiety.

The secondary outcome is to estimate the association of independent variables, including age, sex, educational level, occupation, duration of illness since diagnosis, and duration of hemodialysis, with the level of anxiety and depression among CKD patients.

Statistical analysis

Data were entered and analyzed using the Statistical Package for Social Scientists (SPSS) software version 20 (SPSS Inc., Chicago, IL, USA). Descriptive statistics were presented as frequencies and percentages for categorical variables and as a measure of central tendency (median) and a measure of dispersion (minimum-maximum) for continuous variables. Associations between independent variables (age, sex, education level, occupation, number of dialysis procedures per week, etc.) and the outcome measure (borderline group/cases of anxiety/depression versus normal group, which is the reference group) were performed using the chi-squared test for categorical variables and using the Kruskal-Wallis test for continuous variables. Multivariate analysis was performed using binary logistic regression analysis to test factors independently associated with depression/anxiety among participants. To perform this, the groups of participants were collapsed to only two groups (normal/borderline group, which is the reference, and case group). The P-value was set at ≤0.05.

## Results

Sociodemographic characteristics of the participants

A total of 122 CKD patients were recruited to participate in the study. Table 1 shows that 43.4% of the participants were males and 56.6% were females. Regarding their education, 28.7% of the patients were illiterate, 26.2% had completed high-school education, and 23% held a university degree. More than half of the participants (64.8%) were unemployed. In this study, the median age (in years) was 51.5 years (range: 18-89 years). The median duration of illness since diagnosis was 4 years (range: 0.2-39 years), and the median duration since they started hemodialysis was 3 years (range: 0.1-20 years).

**Table 1:** Sociodemographic characteristics of the participants.

Sociodemographic Variables		Frequency (n = 122)	Percentage
Gender	Male	53	43.4%
	Female	69	56.6%
Education	Illiterate	35	28.7%
	Primary	15	12.3%
	Middle	12	9.8%
	High	32	26.2%
	Bachelor	28	23.0%
Employment	Unemployment/housewife	79	64.8%
	Skillful worker	4	3.3%
	Unprofessional worker	10	8.2%
	Professional worker	13	10.7%
	Own business	4	3.3%
	Retired	12	9.8%

Prevalence of anxiety and depression among CKD patients on hemodialysis

**Figure 1:**
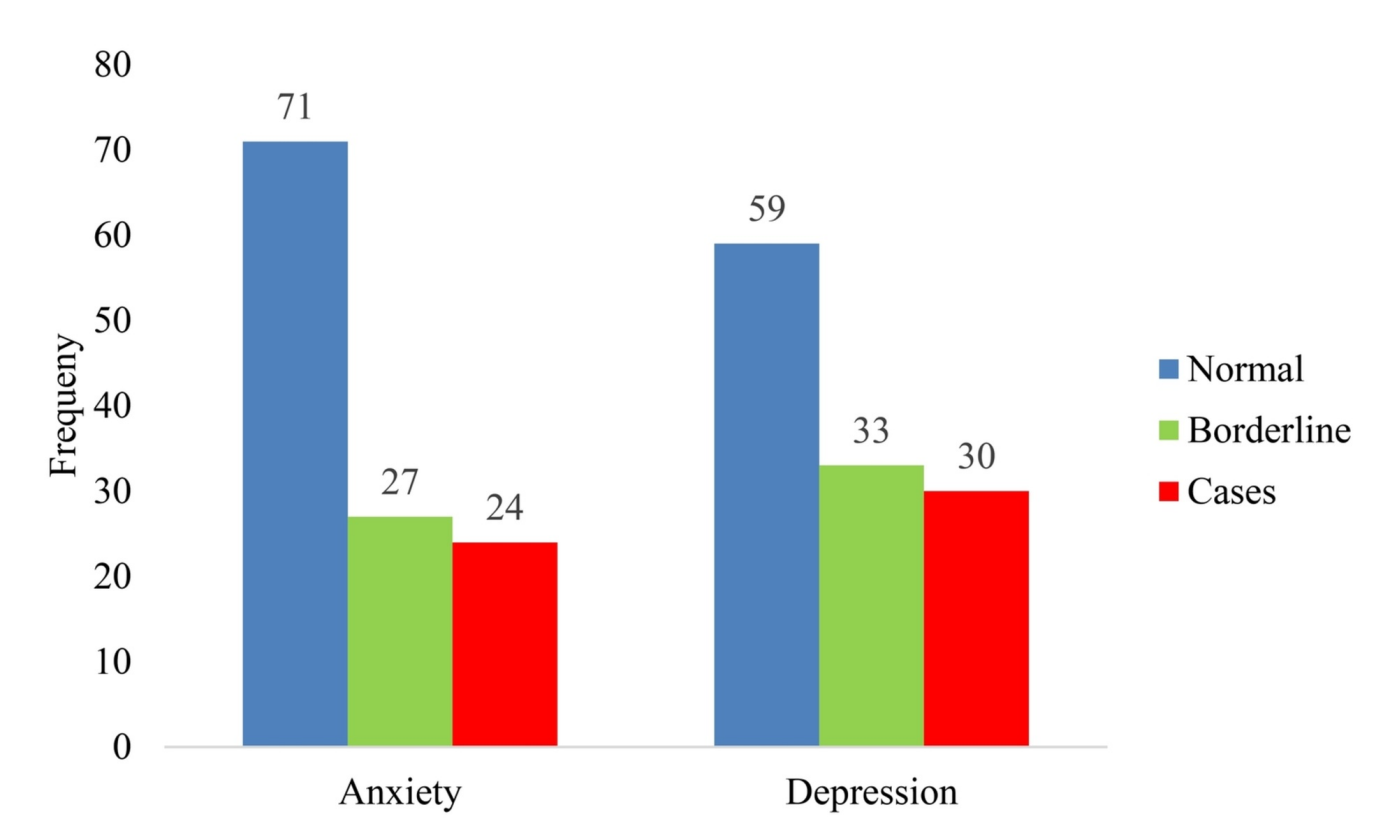
Prevalence of anxiety and depression among the participants.

Figure 1 demonstrates that 71 (58.2%) of the patients were not experiencing anxiety, 27 (22.1%) were considered borderline, and only 24 (19.7%) showed symptoms of anxiety. As regards frequency of depression, 59 (48.4%) of the participants were not experiencing depression, 33 (27%) were considered borderline, and only 30 (24.6%) showed symptoms of depression.

Factors independently associated with anxiety and depression among the participants

Table 2 shows a logistic regression analysis that indicates that none of the factors is independently associated with anxiety. It also demonstrates that only the age of the participant was independently associated with depression among CKD patients (odds ratio: 1.040; 95% CI: 1.004 -1.076; P = 0.027).

**Table 2:** Factors independently associated with anxiety and depression among the participants. OR, odds ratio; CI, confidence interval

Dependent Variables	Independent Variables	Significance Probability	OR	95% CI for OR	
				Lower	Upper
Anxiety	Age	0.103	1.031	0.994	1.070
	Gender	0.054	0.288	0.081	1.022
	Duration of illness since diagnosis	0.980	1.002	0.879	1.141
	Duration of hemodialysis	0.429	1.066	0.910	1.250
	Education level	0.116	2.960	0.766	11.433
Depression	Age	0.027	1.040	1.004	1.076
	Gender	0.773	0.852	0.286	2.536
	Duration of illness since diagnosis	0.634	0.964	0.828	1.122
	Duration of hemodialysis	0.300	1.095	0.922	1.301
	Education level	0.955	1.034	0.325	3.285
	Employment	0.373	0.580	0.175	1.925

## Discussion

CKD is considered one of the most disabling diseases in the world, with a global prevalence rate of 8-16% in 2013 and 11-13% in 2016 [3,11]. Psychiatric disorders usually co-exist with most chronic illnesses and especially with CKD [12]. In this study, the frequencies of depression and anxiety among CKD patients are 24.6% and 19.7%, respectively. Female gender was found to be significantly associated with anxiety (P = 0.04), and older age was significantly associated with depression among the participants (P = 0.003).

Kimmel et al demonstrated that the frequencies of depression and anxiety among patients with CKD or end-stage renal disease (ESRD) are significantly higher than among those with other chronic diseases such as ischemic heart disease, cerebrovascular disease, and peptic ulcer disease [13].

Previous studies established depression as the primary mental health problem of patients with CKD. The prevalence of depression among patients who have CKD is estimated to be between 20% and 30%, which corresponds to the results of this study: 24.6% of the patients in this study were found to have depression (and 27% to have borderline depression). On the other hand, it has been demonstrated that there are increasing levels of anxiety among patients with CKD. A previous study estimated the prevalence rate of anxiety in patients with CKD to be 12% to 52%; this corresponds to the results of this study, which found that 19.7% of patients had anxiety and 22.1% had borderline anxiety [8].

However, a cross-sectional survey conducted in Pakistan in 2013 that used the same HADS questionnaire estimated a higher prevalence of anxiety and depression among patients on hemodialysis: 57.30% of their sample had depression and 42.69% had anxiety disorders [14]. Another study found that depressive disorders were diagnosed in 78.5% of patients on dialysis [15]. However, these discrepancies in the frequency of depression may be attributed to differences in population and the smaller sample size of patients in this study, in addition to socioeconomic differences between the populations.

Yet, a cross-sectional study conducted in Saudi Arabia in 2017 showed that 70% of patients with kidney failure had depression at varying levels. That study found that 28% had mild depression, 26% had moderate depression, 8% had severe depression, and 7% had very severe depression. Among the demographic profile of the patients, socioeconomic status and marital status were found to be associated with depression among renal failure patients (P < 0.05) [16].

On the other hand, multiple studies have not found any significant relationship between depression and anxiety and patients with CKD on hemodialysis or peritoneal dialysis. Stasiak et al estimated the “prevalence of anxiety and depression and its comorbidities in patients with CKD on hemodialysis and peritoneal dialysis” using more than one scale for anxiety and depression. For hemodialysis, the Beck Depression Inventory scale (BDI) showed that 22.6% of patients had depression, and the HADS questionnaire showed 9.3% had depression, whereas anxiety was found in 25.7% of patients on the Beck Anxiety Scale (BAI) and 11.7% in the HADS questionnaire. In the peritoneal dialysis group, 29.6% of patients had depression according to the BDI and 14.8% had it according to the HADS questionnaire, whereas 11.1% of patients had anxiety according to the BAI and none had anxiety according to the HADS questionnaire [17].

Depression and anxiety are strongly dependent on the patient’s sociodemographic data such as age, gender, and level of education. Among our participants, females had a significantly higher anxiety level than males (P < 0.05) in comparison with an insignificant association between female sex and depression level. This finding is consistent with the findings of many previous studies of patients with CKD. Several studies have shown that females usually tend to be more anxious with more suicidal thoughts than males. Theofilou studied depression and anxiety in patients with chronic renal failure. A total of 86 (59.7%) males and 58 (40.3%) females were involved in the study, the results of which showed that females had significantly higher scores than males in trait anxiety measured by the State-Trait Anxiety Inventory II [18]. Hou et al studied the factors associated with depression and anxiety in patients with ESRD who are receiving maintenance hemodialysis. A total of 81 patients were included in the study. Using the self-rating anxiety scale and depression self-assessment scale questionnaires, the results showed that female patients were more likely to develop anxiety, whereas males were more likely to show depressive symptoms [19]. Another recent study conducted in Korea involving 973 subjects aged ≥65 years found that the prevalence of depression in elderly women is higher than in men regardless of CKD stage [20]. Worldwide, it has been frequently shown that women are at higher risk for depression than men, and the female-to-male ratio for depression is approximately 2:1 [21]. Insignificant results of being female and high risk of depression level among our study participants could be modified and achieved by having a larger sample size with hemodialysis multicenter involvement. In addition to the previously mentioned study, female patients tend to develop anxiety, whereas males are more likely to show depressive symptoms.

Regarding age and duration of hemodialysis, many studies have found that older patients are more likely to experience depressive and anxiety symptoms than the other populations studied, as older people are more likely to fall behind on social activities and become socially isolated and depressed. A cross-sectional study conducted in Russia involving 1,047 hemodialysis patients found that advancing age is a predictive factor for a low mental component score along with an increased level of depression and anxiety [22]. Another study confirmed that CKD patients aged >60 years are at higher risk of depression than young patients [23]. This is in agreement with the findings in this study, in which older age was the only factor found to be associated with depression.

With regard to the duration of the dialysis, one previous study suggested that depression and anxiety run different courses in hemodialysis patients. Patients who remained depressed after 16 months of follow-up showed a decrease in quality of life and higher levels of depression; moreover, the prevalence of anxiety associated with depression was higher after 16 months of follow-up [24] Nevertheless, there were no significant results between prevalence rates of anxiety and depression among the participants in this study. Similar findings were also demonstrated in other studies [25].

As regards the educational level of the patient, this study did not show any significant association between the educational level of the participants and anxiety and depression. However, some studies have shown that patients with low educational level and those who are unemployed are at high risk for developing anxiety and depression, presumably due to low socioeconomic status [26]. Another study looked at anxiety and depression in CKD patients in relation to their years of education and reported that patients with less than nine years of education experience more anxiety and depression than the group who had more than nine years of education (P = 0.01 for anxiety and severe depression) [18].

Considering the employment status, a study using the Hamilton Depression Rating Scale showed that 52 of the unemployed patients with chronic renal disease had mild depression, 45 patients had moderate depression, 16 patients had severe depression, and 10 patients had very severe depression. For employed patients, mild depression was found in 12 patients, moderate depression in 13 patients, severe depression in 2 patients, and very severe depression in 5 patients. The P value was non-significant for this research [16]. This agrees with the findings of the current study that proved that employment is not significantly associated with either anxiety or depression.

Nevertheless, knowing that there is a high prevalence of anxiety and depression among patients with CKD is of serious concern and demands a serious and systemic routine screening for these disorders among patients with CKD on hemodialysis, which should be performed in conjunction with providing appropriate interventions. We strongly recommend further research in order to understand how anxiety and depression affect patients with CKD and what are the best interventions to help manage these psychiatric illnesses among those patients.

The main limitation of the study was that only one governmental medical center at Al-Madinah was included. Although this is the largest center for CKD patients in Saudi Arabia, further research that includes other governmental and private centers is needed.

## Conclusions

Depression and anxiety disorders are prevalent among CKD patients who are on hemodialysis: 19.7% of our participants were found to have anxiety and 24.6% were found to have depression. Gender was the only categorical variable associated with anxiety. Meanwhile, older age was found to be significantly associated with depression among the participants. Therefore, examination of these patients for mood disorders in order to achieve early diagnosis and management is needed to improve their quality of life and prevent adverse outcomes. These warrants raising awareness of planning and implementing screening programs for mood disorders among high-risk CKD patients in order to properly manage identified cases.
